# Proteomic Analysis of the Vitreous following Experimental Retinal Detachment in Rabbits

**DOI:** 10.1155/2015/583040

**Published:** 2015-11-18

**Authors:** Nakul Mandal, Geoffrey P. Lewis, Steven K. Fisher, Steffen Heegaard, Jan U. Prause, Morten la Cour, Henrik Vorum, Bent Honoré

**Affiliations:** ^1^Department of Ophthalmology, Lund University, 22185 Lund, Sweden; ^2^Neuroscience Research Institute, University of California, Santa Barbara, Santa Barbara, CA 93106-5060, USA; ^3^Department of Molecular, Cellular and Developmental Biology, University of California, Santa Barbara, Santa Barbara, CA 93106-9625, USA; ^4^Department of Ophthalmology, University of Copenhagen, 2600 Glostrup, Denmark; ^5^Eye Pathology Institute, University of Copenhagen, 2100 Copenhagen, Denmark; ^6^Department of Ophthalmology, Aalborg University Hospital, 9000 Aalborg, Denmark; ^7^Department of Biomedicine, Aarhus University, 8000 Aarhus, Denmark

## Abstract

*Purpose*. The pathogenesis of rhegmatogenous retinal detachment (RRD) remains incompletely understood, with no clinically effective treatment for potentially severe complications such as photoreceptor cell death and proliferative vitreoretinopathy. Here we investigate the protein profile of the vitreous following experimental retinal detachment using a comparative proteomic based approach.* Materials and Methods*. Retinal detachment was created in the right eyes of six New Zealand red pigmented rabbits. Sham surgery was undertaken in five other rabbits that were used as controls. After seven days the eyes were enucleated and the vitreous was removed. The vitreous samples were evaluated with two-dimensional polyacrylamide gel electrophoresis and the differentially expressed proteins were identified with tandem mass spectrometry.* Results*. Ten protein spots were found to be at least twofold differentially expressed when comparing the vitreous samples of the sham and retinal detachment surgery groups. Protein spots that were upregulated in the vitreous following retinal detachment were identified as albumin fragments, and those downregulated were found to be peroxiredoxin 2, collagen-I*α*1 fragment, and *α*-1-antiproteinase F.* Conclusions*. Proteomic investigation of the rabbit vitreous has identified a set of proteins that help further our understanding of the pathogenesis of rhegmatogenous retinal detachment and its complications.

## 1. Introduction

Rhegmatogenous retinal detachment (RRD) is characterized by the accumulation of subretinal fluid between the neurosensory retina and retinal pigment epithelium following the formation of a retinal break [1]. The pathogenesis of RRD is complex and incompletely understood, involving age-related and/or inherited structural and molecular changes of the vitreous extracellular matrix and vitreoretinal interface, and the process of posterior vitreous detachment [2]. The annual incidence of the condition has been estimated at 12.05 per 100,000, and although primary surgical reattachment is successful in the great majority of cases, photoreceptor cell death, subretinal fibrosis, and proliferative vitreoretinopathy (PVR) continue to be significant causes of reduced visual outcomes [3, 4].

PVR involves the proliferation and migration of various cell types including retinal pigment epithelial (RPE) cells, Müller cells, inflammatory cells, and hyalocytes, which contribute to the formation of vitreal and periretinal membranes that can impede photoreceptor regeneration following surgical reattachment and cause tractional retinal detachment. The postulated epithelial-to-mesenchymal transition of RPE cells and Müller cell activation and growth onto the retinal surfaces are believed to be pivotal events in PVR [4, 5]. It appears that the exposure of such cells to the vitreous and associated growth factors as a result of RRD significantly contributes to the pathogenesis of PVR, though the basic cause as well as a clinically effective therapeutic approach for this condition remains elusive [5, 6].

Proteomics studies proteins on a large scale in pursuit of a global and integrated view of disease processes at the protein level, which may potentially lead to the identification of novel biomarkers and therapeutic targets useful in clinical practice [7–11]. RRD would likely be associated with alterations in the proteomic profiles of both the retina and vitreous. Indeed, we initially undertook the first such retinal study from which a number of potentially important proteins were identified [12]. The present study extends the proteomic investigation to the vitreous of this rabbit model of retinal detachment, building upon previous such analyses of human vitreous [13–16], in order to add further knowledge of the underlying pathophysiology [10, 17].

## 2. Materials and Methods

### 2.1. Retinal Detachment Surgery

**Figure 1:**
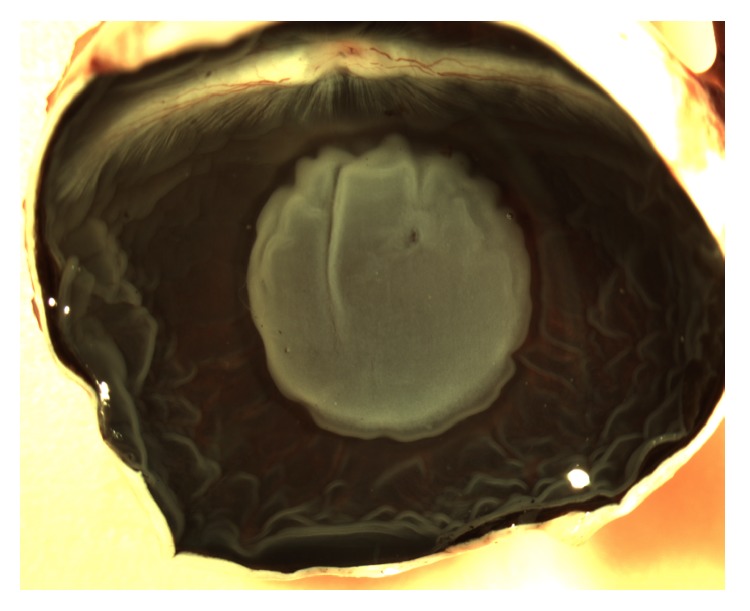
Appearance of the retinal detachment in the rabbit eye at seven days. The area of detached retina beneath the medullary rays appears grey and is surrounded by the darker attached retina. The detached retina contains a small hole where the micropipette was inserted. The retinal folds in the periphery occurred during the removal of the anterior structures.

Inferior retinal detachment was created in the right eyes of six New Zealand red pigmented rabbits. The eyes were normal with no evidence of disease on examination. Combined injections of xylazine (6.7 mg/kg) and ketamine (33.3 mg/kg) were administered intramuscularly to induce anesthesia and analgesia. The pupils were dilated with topical drops of atropine and tropicamide (1% solutions). A pipette tip, with an external diameter of approximately 100 *μ*m, was inserted into the eye through a pars plana incision. Sodium hyaluronate (Healon, 0.25% in a balanced salt solution; Pharmacia, Piscataway, NJ) was infused via a glass pipette between the neurosensory retina and retinal pigment epithelium. Healon was necessary to prevent spontaneous retinal reattachment, and 0.25% is the most dilute solution that maintains the detachment for extended periods. Approximately 50% of the retina beneath the medullary rays, which included the central retina, was detached (Figure 1). Sham surgery was performed in the right eyes of five other rabbits that were used as controls, which involved surgical entry of the vitreous cavity without disruption of the retina. Scleral incisions were closed with 8-0 nylon suture. Seven days postoperatively the animals were euthanized by the administration of sodium pentobarbital (120 mg/kg; Butler Schein, Dublin, OH) and the eyes enucleated. After removal of the cornea and lens, the associated vitreous of the sham and detached retinas was extracted and immediately snap-frozen in liquid nitrogen within separate vials. There was no gross evidence of blood or other contamination of the vitreous samples at the time of tissue harvesting. The vitreous samples were stored at −80°C until further use.

All of the animal experiments undertaken in this study were in accordance with the standards of the National Institutes of Health Animal Care and Use Committee protocols, the ARVO Statement for the Use of Animals in Ophthalmic and Vision Research, and the guidelines of the Animal Resource Center, University of California, Santa Barbara.

### 2.2. Protein Extraction

The rabbit vitreous samples were homogenized and dissolved in a lysis buffer containing 9 M urea, 2% (v/v) Triton X-100, 2% (v/v) immobilized pH gradient (IPG) buffer (pH 3–10 nonlinear), and 2% (w/v) dithiothreitol (DTT). The total protein content in each vitreous sample was determined with Non-Interfering Protein Assay (Calbiochem, San Diego, CA). The protein samples were stored at −80°C until further use.

### 2.3. Two-Dimensional Gel Electrophoresis

The extracted proteins were first fractionated by isoelectric focusing (IEF) using pH 3–10 nonlinear 18 cm IPG strips (GE Healthcare, Chalfont St. Giles, Buckinghamshire, UK). The IPG strips were rehydrated for 20 h at room temperature in 200 *μ*L lysis buffer each containing 20 *μ*g protein from individual vitreous samples and 150 *μ*L rehydration buffer (8 M urea, 2% (w/v) 3-[(3-cholamidopropyl)dimethylammonio]-1-propanesulfonate (CHAPS), 0.3% (w/v) DTT, and 2% (v/v) IPG buffer), using the Immobiline DryStrip Reswelling Tray (GE Healthcare). The IEF was undertaken on a Multiphor II Electrophoresis System (GE Healthcare) at 500 V for 5 h and 3500 V in two steps for 5 h and 9.5 h in a gradient mode at 17°C with the use of a MultiTemp III Thermostatic Circulator (GE Healthcare). Before the second-dimension sodium dodecyl sulfate (SDS) polyacrylamide gel electrophoresis (PAGE), the IPG strips were equilibrated firstly for 10 min with gentle agitation in 20 mL of equilibration solution (0.6% (w/v) Tris-HCl, pH 6.8, 6 M urea, 30% (v/v) glycerol, 1% (w/v) SDS, and 0.05% (w/v) DTT) and secondly using 4.5% (w/v) iodoacetamide and bromophenol blue. The IPG strips were then transferred to 12% polyacrylamide gels for electrophoresis, which was performed at a maximum voltage of 50 V for approximately 20 h to separate the proteins vertically on the basis of molecular mass.

### 2.4. Protein Staining

The two-dimensional (2D) gels were silver stained using a protocol optimized for protein identification with mass spectrometry [18]. In brief, the gels were fixed overnight in 50% (v/v) ethanol, 12% (v/v) acetic acid, and 0.0185% (v/v) formaldehyde. The gels were washed 3 times for 20 min in 35% (v/v) ethanol and pretreated for 1 min in 0.02% (w/v) Na_2_S_2_O_3_·5H_2_O. They were then rinsed in water and stained for 20 min in 0.2% (w/v) AgNO_3_ and 0.028% (v/v) formaldehyde. Following further rinsing with water, development was undertaken for approximately 3 min in 6% (w/v) Na_2_CO_3_, 0.0185% (v/v) formaldehyde, and 0.0004% (w/v) Na_2_S_2_O_3_·5H_2_O. The development was arrested in a fixative solution of 40% (v/v) ethanol and 12% (v/v) acetic acid. The 2D gels were then dried between cellophane sheets and sealed in plastic envelopes.

### 2.5. Image Analysis

Silver stained 2D gels were scanned on a GS-710 Calibrated Imaging Densitometer (Bio-Rad, Hercules, CA) using the Quantity One program (Bio-Rad), and the PDQuest software (Bio-Rad) was used to define, quantify, and match the protein spots on each of the 2D gels. All well-defined protein spots that were at least twofold (Mann-Whitney *U* test, *p* < 0.05) differentially expressed between the sham and retinal detachment vitreous groups were selected for identification with nanoliquid chromatography-electrospray ionization tandem mass spectrometry (LC-MS/MS).

### 2.6. Protein Identification

The 2D gels were removed from their plastic envelopes and rehydrated in water. The selected protein spots were carefully excised from the gels with a scalpel and subjected to in-gel digestion with Trypsin Gold (Mass Spectrometry Grade; Promega, Madison, WI). The peptide samples that were obtained were analyzed by LC-MS/MS as previously described [12]. In brief, peptides generated by trypsin digestion were separated on an inert nano-LC system (LC Packings, San Francisco, CA) connected to a Q-TOF Premier mass spectrometer (Waters, Milford, MA). The MassLynx 4 SP4 (Waters) was used to obtain spectra and the raw data was processed using ProteinLynx Global Server 2.1 (Waters). The processed data were used to search the total part of the Swiss-Prot database using the online version of the Mascot MS/MS Ions Search facility (Matrix Science, Ltd.). The search was undertaken with doubly and triply charged ions with up to two missed cleavages, a peptide tolerance of 50 ppm, one variable modification, carbamidomethyl-C, and a MS/MS tolerance of 0.05 Da. Contaminating peptides such as trypsin, keratin, bovine serum albumin, and all peptides originating from previous samples were disregarded. At least one “bold red” (Matrix Science Ltd., http://www.matrixscience.com/) peptide match was required in the search for protein hits. Individual peptide ions scores above approximately 36 indicated identity or extensive homology giving a less than 5% probability that the observed match was a random event. All peptides for the protein hits are reported (Table 1).

### 2.7. Western Blotting

In each case three micrograms of vitreous sample protein was separated on Novex 10–20% gradient Tris-Glycine polyacrylamide gels (Invitrogen Corporation, Carlsbad, CA) and subsequently transferred to nitrocellulose Hybond-C Extra membranes (GE Healthcare). The membranes were blocked overnight with 5% skimmed milk in 80 mM Na_2_HPO_4_, 20 mM NaH_2_PO_4_, 100 mM NaCl, and 0.05% Tween 20 buffer, pH 7.5. Membranes were incubated with anti-albumin (Genway Biotech, CA, USA; 1 : 5000) and anti-peroxiredoxin 2 (Abcam, Cambridge, UK; 1 : 200). No suitable antibodies were commercially available for the rabbit F isoform of *α*-1-antiproteinase or the rabbit collagen-I*α*1 fragment that was identified with LC-MS/MS. Following washing, the membranes were further incubated with appropriate horseradish peroxidase-conjugated secondary antibodies: P0163 sheep and P0260 mouse (both 1 : 1000; DAKO, Glostrup, Denmark). Proteins were visualized with the enhanced chemiluminescence system (GE Healthcare) and imaging system (Fujifilm LAS-3000, Tokyo, Japan).

## 3. Results

### 3.1. 2D-PAGE Analysis

**Figure 2:**
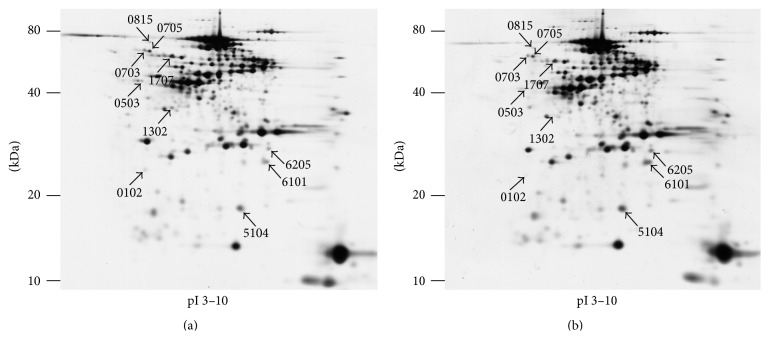
Representative 2D-PAGE images of proteins fractionated from rabbit vitreous. Ten protein spots (arrows) were found to be significantly and at least twofold differentially expressed between the sham (a) and detached (b) vitreous.

Up to approximately 340 protein spots were clearly resolved on each of the 11 2D gels. Ten protein spots were found to be significantly and at least twofold differentially expressed between the sham and detachment vitreous groups (Figure 2). Three protein spots were upregulated and seven spots were downregulated.

### 3.2. LC-MS/MS Analysis

From the three upregulated protein spots, two were identified as fragments of albumin (spots 5104 and 6101), whilst spot 6205 could not be identified. Four of the seven downregulated protein spots were identified as fragment of collagen-I*α*1 (spot 0503), *α*-1-antiproteinase F (spots 0703 and 1707), and peroxiredoxin 2 (spot 0705). Protein spots 0102, 0815, and 1302 could not be identified (Figure 2; Table 1).

### 3.3. Western Blot Analysis

**Figure 3:**
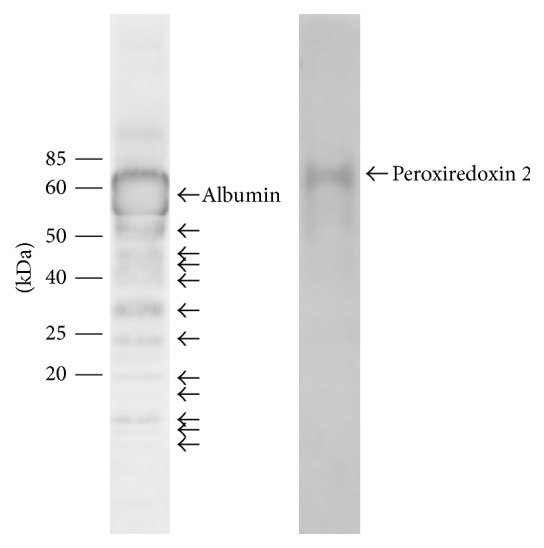
Western blot analysis of sham rabbit vitreous developed with anti-albumin and anti-peroxiredoxin 2. Labeled arrows correspond to the respective full length proteins. The unlabeled arrows for the lower molecular mass bands on the anti-albumin blot may indicate the specific cleavage fragments, which correspond with some of the differentially expressed 2D-PAGE protein spots.

Western blotting developed with anti-albumin showed a heavy band at approximately 60 kDa, which is likely to represent the full length protein, whilst multiple bands below this suggest the presence of several fragments, some of which may correspond with those identified with the 2D-PAGE analysis (Figure 3, left). Peroxiredoxin 2 has a deduced molecular mass of approximately 22 kDa. However, spot 0705 containing peroxiredoxin 2 migrates with a molecular mass around 60 kDa with 2D-PAGE (Figure 2), and this size was verified by western blot analysis (Figure 3, right). Though a single and specific band was achieved with anti-peroxiredoxin 2, western blotting could not be reliably used for quantification due to a weak signal near the detection limit and variable background reaction.

## 4. Discussion

### 4.1. Albumin

Analysis with 2D-PAGE revealed fragments of albumin to be upregulated in the vitreous following retinal detachment. Albumin is the most abundant protein in plasma, aqueous, and vitreous humor, where in the latter it constitutes around 60–70% of total protein [19–21]. Serum proteins such as albumin are present in the aqueous and vitreous humor at a relatively lower level compared to the vascular circulation from where they may have in part originated [21, 22]. Western blot analysis showed an intense band at approximately 60 kDa corresponding with the full length albumin protein, with multiple lower molecular mass bands that are likely its fragments. Increase of albumin and its fragments may signify increased proteolysis and the passage of albumin into the vitreous. Indeed, the breakdown of the blood-retinal barrier that occurs with retinal detachment has also been implicated in the increase of other such proteins in the vitreous [12, 23–28]. It is also possible that albumin in the vitreous may arise from de novo synthesis in the retina, similar to the reported increased gene and protein expression of albumin in the corneal epithelium during wound healing [29, 30].

Extraocular albumin is known to have diverse and important functions, which include maintenance of colloid osmotic pressure, transport of biomolecules, and inactivation of toxins through intermolecular binding [31, 32]. Albumin can also act as an antioxidant by scavenging reactive oxygen species and sequestration of metal ions and has anti-inflammatory and apoptotic regulatory abilities [32–35]. Vitreal albumin has been proposed to transport long chain fatty acids into the lens for biosynthesis of lenticular lipids [21, 31, 36]. Indeed, albumin is likely to have many such important roles in the eye, which requires further investigation.

### 4.2. Peroxiredoxin 2

In the present study we observed peroxiredoxin 2 to have a molecular mass above 60 kDa using both 2D-PAGE and western blot analyses. However, the predicted molecular mass of the peroxiredoxin family of proteins is approximately 22 kDa–31 kDa. This variation may represent the well-studied property of these proteins to undergo oligomerization, which can be promoted by a number of factors including overoxidation of cysteine residues of peroxiredoxin [37]. Although the present experiments were conducted in standard reducing conditions that aim to break cysteine bonds, we obtained a band well above 20 kDa. This is in keeping with another study, which also showed some peroxiredoxin 2 western blot bands appearing at molecular mass much higher than 20 kDa that was suggested to result from oligomerization or posttranslational modification [38]. Our finding could also represent a novel alternative splicing variant of peroxiredoxin 2, as reported for peroxiredoxin 5 [39].

The peroxiredoxins are a group of ubiquitous antioxidant proteins that currently comprise six members in mammals [40]. These proteins are primarily found at high levels intracellularly, mainly within the cytosol, but are also present in the mitochondria, peroxisomes, and nuclei, and they may be exported [37]. Furthermore, presence of peroxiredoxin 2 has been shown in plasma, not only as a result of hemolysis but also possibly by secretion from the T lymphocytes [41, 42]. These multifunction enzymes act as antioxidants by using redox active cysteines for the reduction and degradation of hydrogen peroxide, peroxynitrite, and organic hydroperoxides [37, 43]. Oxidative stress is thought to result from an imbalance between reactive oxygen species production and antioxidant ability and is recognized to be an important factor in the pathogenesis of a number of age-related and neurodegenerative diseases, which include age-related cataract, age-related macular degeneration, glaucoma, diabetic retinopathy, retinal detachment, and PVR [44–47]. Indeed, the present study showed a decrease in the vitreal levels of peroxiredoxin 2 following retinal detachment. This may be in keeping with reported reductions in the levels of other members of the antioxidant defense system such as glutathione and ascorbic acid both in vitreal and in blood samples of patients suffering from PVR [44, 45]. Furthermore, apart from their role as antioxidants, the peroxiredoxins can affect a diverse range of biological processes that include cellular proliferation, differentiation, and apoptosis by influencing signal transduction pathways that employ hydrogen peroxide as a secondary messenger [43, 48]. Recent studies on tears from patients with glaucoma have also identified peroxiredoxin 1 as having a possible involvement in inflammation [49, 50]. Indeed, peroxiredoxin 2 and other members of this family of proteins are liable to have a significant role in the pathophysiology of retinal detachment.

### 4.3. Collagen-I*α*1

A fragment of collagen-I*α*1 was identified in the vitreous of the rabbit; however, type I collagen has not previously been identified as a natural component of the mammalian vitreous and is rather known to be a constituent of early PVR membranes [51–53] and retinal blood vessels [54, 55]. A mixture of type II, IX, and V/XI hybrid collagen fibrils, which are separated out mainly by water and ions attracted to hyaluronan, characterizes the vitreous body [56]. Collagen, possibly with the aid of adhesive-like intermediate molecules, may provide the basis of vitreoretinal adhesion by connecting the vitreous with the retinal inner limiting membrane (ILM). This attachment is extremely strong in the vitreous base since the fibrils pass through the ILM to merge into underlying collagen networks and crypts [2, 56, 57]. Collagen is also a significant component of both epiretinal and subretinal PVR membranes [53, 58], and type I collagen is recognized to be a principal constituent during their early development [51–53]. The presence of collagen in the subretinal space, a place normally devoid of this protein, suggests that certain cells, particularly the RPE and Müller cells associated with membranes, are able to synthesize collagen under certain pathological conditions such as retinal detachment and PVR [58–61]. However, the present analysis suggests collagen-I*α*1 fragment to be found in sham vitreous, which furthermore showed a decreased concentration following retinal detachment that may indicate perturbed proteolytic activity. Matrix metalloproteinases (MMP) and other proteolytic enzymes that are able to degrade and remodel vitreal collagen have been found to be increased with RRD and PVR [62–65], which could be in keeping with the decrease in *α*-1-antiproteinase shown in the present study. Further studies will be necessary to confirm the source and nature of collagen-I*α*1 in the vitreous and the possible mechanisms of collagen fragmentation that may be an important feature of vitreous liquefaction and RRD [2, 62, 66].

### 4.4. Alpha-1-Antiproteinase

2D-PAGE showed *α*-1-antiproteinase (also called *α*-1-antitrypsin or *α*-1-proteinase inhibitor) at two closely positioned spots, which were largely in keeping with their predicted molecular mass but differing by their charge. Currently, four isoforms of *α*-1-antiproteinase have been identified in the rabbit, termed F, S1, S2, and E, which is a similar picture to the multiple variants identified in humans [67, 68]. Alpha-1-antiproteinase is an acute phase protein and archetypal member of the superfamily of serine protease inhibitors (serpin), which are involved in a wide range of biological processes that includes inflammation, angiogenesis, blood coagulation, ECM remodeling, and tumor suppression [69]. This protein has the ability to inhibit a large number of serine proteases though its principle target is neutrophil elastase [68]. Indeed, *α*-1-antiproteinase originally received much attention because its deficiency increases the risk of a variety of clinical conditions, such as chronic obstructive pulmonary disease, which can result from unrestrained elastase activity.

We found the F isoform of rabbit *α*-1-antiproteinase to be downregulated in the vitreous following retinal detachment. The F isoform of *α*-1-antiproteinase is the only one of the rabbit isoforms so far identified that has been shown to have the oxidizable methionine residue site that is present in human *α*-1-antiproteinase [67]. The oxidation of methionine to methionine sulfoxide, which can occur during episodes of inflammation as a result of oxygen-free radicals secreted by leucocytes, has an inhibiting effect upon *α*-1-antiproteinase function. This process is thought to enhance the ability of proteinases such as elastase to locally degrade tissue debris that occurs at sites of inflammation [67, 68].

Alpha-1-antiproteinase is primarily produced in the liver and circulated to the rest of the body tissues via the blood; however, extrahepatic sites of its synthesis have been identified, which include blood monocytes, alveolar macrophages, bronchial and gastrointestinal epithelial cells, and the cornea [70–73]. The protein has also been localized to the tear film, aqueous humor, and vitreous, where in the latter a phosphorylated form of *α*-1-antiproteinase has been suggested as a potential biomarker of idiopathic macular hole and rhegmatogenous retinal detachment [73–75]. It has been postulated that one of the main functions of corneal *α*-1-antiproteinase is to protect against the damaging effects of neutrophil elastase produced during corneal inflammation [73], and it may be expected that a similar role in addition to others is applicable to vitreal *α*-1-antiproteinase, though this requires further investigation.

## 5. Conclusion

This proteomic investigation of the rabbit vitreous has identified a set of proteins that assist our understanding of the pathogenesis of rhegmatogenous retinal detachment and its complications. Further studies will be necessary to clarify the role of these proteins. Certain proteins, such as those of low abundance and at the extremes of molecular mass, together with membrane proteins, can be difficult to resolve and detect using the 2D-PAGE technique. Therefore, complementary proteomic methods such as gel-free mass spectrometry should be considered in future work in order to help address these limitations.

## Figures and Tables

**Table 1:** Mass spectrometric identification of the 2D-PAGE protein spots differentially expressed in the rabbit vitreous.

Spot number	Protein name (full chain length)	Peptide sequence (amino acid numbers)	Mascot Ions score	Theoretical pI; Mr kDa	Fold change RD: sham	Biological processes
5104	*Albumin* (608) fragment	CCSESLVDR (500–508) TVVGEFTALLDK (570–581) EACFAVEGPK (589–598)	52 14 28	5.67; 66.5	2.62	Transport; cellular response to starvation; maintenance of mitochondrial location; negative regulation of apoptosis; hemolysis of symbiont of host erythrocytes

6101	*Albumin* (608) fragment	ACVADESAANCDK (76–88) DTYGDVADCCEK (106–117)	39 7	5.67; 66.5	3.13	

0503	*Collagen type Iα1* (1463) fragment	GETGPAGPAGPIGPVGAR (1066–1083)	61	9.20; 94.4	0.46	Blood vessel development; collagen biosynthetic process; collagen fibril organization; leukocyte migration; platelet activation; positive regulation of cell migration, epithelial-to-mesenchymal transition, transcription, and canonical Wnt receptor signaling pathway; protein localization to nucleus; protein transport; visual perception; axon guidance

1707	*α-1-antiproteinase F* (413)	GDTHTQVLEGLK (89–100)	64	5.76; 43.5	0.42	Negative regulation of peptidase activity

0703	*α-1-antiproteinase F* (413)	IVDLVQELDAR (188–198)	70	5.76; 43.5	0.24	

0705	*Peroxiredoxin 2* (198)	TDEGIAYR (120–127)	43	5.67; 21.8	0.30	Response to oxidative stress; regulation of hydrogen peroxide metabolic process; removal of superoxide radicals; positive regulation of blood coagulation; activation of MAPK; respiratory burst involved in inflammatory response; antiapoptosis; regulation of apoptotic process; negative regulation of lipopolysaccharide mediated signaling pathway; negative regulation of NF-KappaB transcription factor; T cell proliferation; homeostasis of number of cells

Protein spots 6205, 1302, 0815, and 0102 with fold changes of 2.65, 0.28, 0.27, and 0.18, respectively, could not be identified. Individual Mascot Ions scores greater than approximately 36 indicate identity or extensive homology (*p* < 0.05). Biological processes were taken from the Gene Ontology Consortium (http://geneontology.org/). RD represents retinal detachment.
